# Factor structure of the Hospital Anxiety and Depression Scale (HADS) in German coronary heart disease patients

**DOI:** 10.1186/1477-7525-3-15

**Published:** 2005-03-16

**Authors:** Jürgen Barth, Colin R Martin

**Affiliations:** 1 University of Freiburg – Institute of Psychology, Department of Rehabilitation Psychology, 79085 Freiburg, Germany; 2 The Nethersole School of Nursing, Faculty of Medicine, The Chinese University of Hong Kong, Esther Lee Building, Chung Chi College, Shatin, New Territories, Hong Kong, China

## Abstract

**Background:**

Depression and anxiety in patients with coronary heart disease (CHD) are associated with a poorer prognosis. Therefore the screening for psychological distress is strongly recommended in cardiac rehabilitation. The Hospital Anxiety and Depression Scale (HADS) is a widely used screening tool that has demonstrated good sensitivity and specificity for mental disorders.

**Methods:**

We assessed mental distress in in-patient cardiac rehabilitation in Germany. The factor structure of the German language version of the HADS was investigated in 1320 patients with CHD. Exploratory factor analysis and confirmatory factor analysis were used to determine the underlying factor structure of the instrument.

**Results:**

Three-factor models were found to offer a superior fit to the data compared to two-factor (anxiety and depression) models. The German language HADS performs similarly to the English language version of the instrument in CHD patients. The German language HADS fundamentally comprises a tri-dimensional underlying factor structure (labelled by Friedman et al. as psychomotor agitation, psychic anxiety and depression).

**Conclusion:**

Despite of clinical usefulness in screening for mental disturbances the construct validity of the HADS is not clear. The resulting scores of the tri-dimensional model can be interpreted as psychomotor agitation, psychic anxiety, and depression in individual patient data or clinical investigations.

## Background

Coronary heart disease (CHD) is of profound interest to health and clinical psychology due to the high levels of anxiety and depression observed in patients following the occurrence of a coronary event [1-5]. CHD was the most leading diagnosis for treatment in hospitals in Germany for men (320,000 patients per year), and, after childbirth and breast cancer, the third reason for in-patient treatment for women (150,000 patients per year) [6]. Most in-patient rehabilitation hospitalizations in Germany for men (about 60,000 per year) were caused by CHD [7,8]. Recent research showed, that at least one in five patients in cardiac rehabilitation suffer from a psychological disorder [9]. Accurate identification of significant anxiety and depression as soon as possible following a cardiac event is essential in order to facilitate delivery of an effective and comprehensive treatment package which takes into account psychological as well as coronary disease symptoms [10]. This is particularly relevant since anxiety and especially depression have been demonstrated to be predictors of mortality in this clinical group [11]. The availability of easy to administer, reliable and valid screening tools would logically be a critical component of a clinical protocol seeking to identify CHD patients with psychological disturbance. A suitable measure would readily identify those patients for whom additional referral to a clinical psychologist or to a liaison psychiatry service would be more appropriate.

A candidate screening tool that has been widely and increasingly used with CHD patients is the Hospital Anxiety and Depression Scale (HADS: [12]), an easily administered 14-item self-report measure comprising 7 anxiety items and 7 depression items from which separate anxiety and depression sub-scale scores are calculated [13]. The HADS was designed to exclude symptoms that might arise from the somatic aspects of illness such as insomnia, anergia, and fatigue, therefore the instrument has been designed for use within the clinical context of general medicine. The HADS has been used for screening purposes in a diverse and broad range of clinical groups [14-24]. A number of investigations have suggested that the HADS is a suitable instrument to accurately assess anxiety and depression in CHD patients [10,17,24-27]. A fundamental assumption underpinning the clinical usefulness of the HADS across a broad range of clinical groups, including CHD, is that the instrument reliably assesses anxiety and depression as two distinct and separable dimensions [28].

On the other hand, recent psychometric evaluations of the HADS in a range of clinical populations have suggested that the proposed factor structure of the instrument may indeed be compromised by the physiological aspects of the disease or by changes in health status [23,29,30]. Conversely, there is accumulating evidence that the fundamental factor structure of the HADS comprises three factors instead of two [24,26,31-33]. The finding that the three-factor structure offers a superior fit to clinical data than the two-factor (anxiety and depression) model formulated as part of the original instrument development by Zigmond and Snaith [12] has implications in terms of the use, scoring and future development of this assessment tool.

Dunbar et al. [32] found a three-factor structure of the HADS in a non-clinical population (for an overview see table 1) and interpreted their findings in relation to the conceptually rich 'tripartite' model proposed by Clark and Watson [34]. Extending these observations to a clinical population, Friedman et al. [33] found a three-factor structure to the HADS in a patient group being treated for major depression, which incidentally, was similar to that observed by Dunbar et al. [32]. Martin and Newell [35] found that Friedman et al.'s [33] three-factor model offered the best-fit to their data examining individuals with significant facial disfigurement compared to competing two-factor models. Martin and Thompson [22] observed a three-factor structure to the HADS in myocardial infarction patients and, in a later study, Martin et al. [26] extended further the findings of both Dunbar et al. [32] and Friedman et al. [33] to myocardial infarction patients finding additional support for the three-factor structure suggested by these researchers to underlie the HADS. A recent study [24] of the psychometric properties of the HADS in Chinese acute coronary syndrome (ACS) patients has established further support for the three-factor structure of the HADS furnishing evidence that the three-dimensional structure of the instrument appears to be consistent across diverse cultures. Caci et al. [31] suggested a three-factor underlying structure to the HADS that represents a modification to the three-factor model identified by Friedman et al. [33] and replicated by Martin et al. [26]. However, Caci et al.'s [31] model was based on a student population and it should be remembered that the presence of significant pathology or physiological change states does impact on the underlying factor structure of this instrument [23,30,36]. The most consistent contemporary observation in terms of the underlying factor structure of this instrument in cardiac patients is strongly indicative that the HADS comprises three underlying dimensions. However, these cardiac studies have used either clinical populations from the United Kingdom [22,26] or from the Far East [24].

**Table 1:** Characteristics of each factor model tested in earlier studies.

**Model, author, year**	**Number of factors**	**Clinical population**	**n**	**Factor extraction method ^#^**	**Allocation of items to scales**
Zigmond & Snaith (1983)	2	Medical	100	No Factor analysis conducted	anxiety: 1, 3, 5, 7, 9, 11, 13 depression: 2, 4, 6, 8, 10, 12, 14

Moorey et al. (1991)	2	Cancer	568	PCA	anxiety: 1, 3, 5, 9, 11, 13 depression: 2, 4, 6, 7, 8, 10, 12, 14

Dunbar et al. (2000)	3	Non-clinical	2,547	CFA	autonomic anxiety: 3, 9, 13 negative affectivity: 1, 5, 7, 11 anhedonic depression: 2, 4, 6, 7,

Friedman et al. (2001)*	3	Depressed	2,669	PCA	psychomotor agitation: 1, 7, 11 psychic anxiety: 3, 5, 9, 13 depression: 2, 4, 6, 8, 10, 12

Razavi et al. (1990)	1	Cancer	210	PCA	All items included

Caci et al. (2003)	3	Non-clinical	195	CFA	anxiety: 1, 3, 5, 9, 13 restlessness: 7, 11, 14 depression: 2, 4, 6, 8, 10, 12

*The three-factors are correlated in this model.

^#^PCA: Principal Components Analysis; CFA: Confirmatory Factor Analysis.

There has been little systematic investigation of the factor structure of the translated HADS in German cardiac patients, though interestingly a German language version of the instrument has been developed in Germany using cardiac patients within the context of the original assumed two-dimensional (anxiety and depression) structure [37]. To date, the HADS has been found to comprise a two-factor structure consistent with the anxiety and depression sub-scales proposed by Zigmond and Snaith [12] in cardiac [38] and non-clinical [39] populations in Germany. However, these large studies did not investigate the possibility that alternative factor models may provide a better explanation of the data.

Identification of a coherent three-factor underlying structure of the HADS has a number of significant implications in terms of the validity of the tool as a screening instrument. Firstly, referral to mental health services could be undermined based on a two-dimensional (anxiety and depression) interpretation of HADS scores in cardiac patients. Secondly, further replication of a three-factor structure of the HADS in a German cardiac population would be valuable in determining if the HADS should be more effectively used as a screening instrument when comprised of three sub-scales in this group. Thirdly, replication of a consistent three-factor structure in the German-translated version of the HADS would provide strong evidence that the three-dimensional structure is implicit to the instrument and not a language-based artifact. Finally, the widespread international use of the HADS provides a compelling rationale to establish the psychometric properties of the instrument not only in broad diagnostic categories, but also across culturally-diverse groups.

The purpose of the present study was to determine whether the three-factor structure of the HADS identified by Martin and colleagues [26] in myocardial infarction patients in the UK and Martin et al. [24] in Chinese ACS patients has the same psychometric properties as that of the German-translated version of the HADS in a cohort of German patients presenting with CHD. The present study addresses two research questions:

1) Do exploratory factor analysis (EFA) and confirmatory factor analysis (CFA) techniques concord to the proposed bi-dimensional structure of the HADS in German CHD patients?

2) Does a three-dimensional factor structure provide a superior fit compared to competing bi-dimensional factor structures?

## Methods

### Design

The study used a cross-sectional design with all measures taken at one observation. The dependent variables were sum scores obtained on the HADS (all items), and the anxiety (HADS-A) and depression (HADS-D) sub-scales. Exploratory factor analysis (EFA) and confirmatory factor analysis (CFA) methods were used to address the research questions using a pooled HADS data set from all patients. Ethical approval for the study was given by the local ethical committee of the University Hospital of Freiburg. Written informed consent was obtained from all participants prior to the commencement of the study.

### Procedure

The study was conducted in three German cardiac rehabilitation hospitals. The patients stay in these hospitals for three to four weeks for a comprehensive cardiac rehabilitation program consisting of medical advice, exercise, patient education, relaxation and psychosocial interventions. All cardiac patients who agreed to take part in the PROTeCD-study (Psychotherapeutic Resource-Orientated Treatment for Cardiac Patients with Depression) were screened for mental distress with the German version of the HADS [37] at admission to hospital. Sociodemographic data were collected by self report and somatic data were reported by physicians at study entry.

### Statistical analysis

#### Exploratory factor analysis

Exploratory factor analysis was performed on the full 14-item HADS using SPSS 12.0 statistical software. The criterion chosen to determine that an extracted factor accounted for a reasonably large proportion of the total variance was based on an eigenvalue greater than 1. A maximum likelihood factor extraction procedure was chosen since this method of factor condensation is consistent with our previous research [22] and is particularly useful for extracting psychologically meaningful factors [40]. An oblimin non-orthogonal factor rotation procedure was chosen [40] due to the possibility that extracted factors are likely to be correlated. Determination of a significant item-factor loading was set at a coefficient level of 0.30 or greater, this level based on a rationale of generating a more complete psychological interpretation of the data set, this being a level consistent with investigators who have used EFA [22,30,36,41].

#### Confirmatory factor analysis

Confirmatory factor analysis was conducted using the Analysis of Moment Structures (AMOS) version 4 [42] statistical software package. Eight models were tested. These were Zigmond and Snaith's [12] original two-factor model, Moorey et al.'s [43] two-factor model, Razavi et al.'s [44] single-factor model, two versions of Clark and Watson's [34] three-factor model as evaluated by Dunbar and colleagues [32], Friedman et al.'s [33] three-factor model and two versions of Caci et al.'s [31] three-factor model. The characteristics and the allocation of the items to the factors in each tested modelare shown in Table 1. For all models, independence of error terms was specified. Factors were allowed to be correlated where this was consistent with the particular factor model being tested. Multiple goodness of fit tests [45] were used to evaluate the eight models, these being the comparative fit index (CFI; [46]), the Akaike information criterion (AIC; [47]), the consistent Akaike information criterion (CAIC; [48]), the normed fit index (NFI; [45]), the goodness of fit index (GFI; [49]) and the root mean squared error of approximation (RMSEA). A CFI greater than 0.90 indicates a good fit to the data [50]. A NFI and GFI greater than 0.90 indicates a good fit to the data [51]. A RMSEA with values of less than 0.08 indicates a good fit to the data [52], while values greater than 0.10 suggest strongly that the model fit is unsatisfactory. The AIC and CAIC are useful fit indices for allowing comparison between models [32]. The Chi-square goodness of fit test was also used to allow models to be compared and to determine the acceptability of model fit. A statistically significant χ^2 ^indicates a significant proportion of variance remains unexplained by the model [45].

## Results

### Participants

1320 patients (1035 male) enrolled in an in-patient cardiac rehabilitation programme in three hospitals in Germany provided complete HADS data sets for analysis. Inclusion criteria for participation in the study was a confirmed diagnosis of CHD; for details see [53]. The patient group comprised patients with a diagnosis of myocardial infarction (N = 666), coronary artery bypass graft (N = 382), percutaneous transluminal coronary angioplasty (N = 303) and unstable angina pectoris (N = 40). It is noted that diagnostic N exceeds total cohort N because many patients will have multiple CHD diagnoses. Patients were required to have had a diagnosis of CHD and had a recent cardiac event (MI, CABG, PTCA) in the past weeks. Female patients (mean age = 62.88; SD = 12.14) were significantly older, (*t*_(401.14) _= 4.14, *p *< 0.001) than male patients (mean age = 59.58; SD = 10.59).

### Descriptive findings

The mean HADS-A sub-scale score was 6.14 (SD = 4.15, range 0–20) and the mean HADS-D sub-scale score was 5.41 (SD = 4.00, range 0–20). Using Snaith and Zigmond's [28] cut-off criteria of HADS-A and HADS-D scores of eight or over, 467 participants (35%) demonstrated *possible *clinically relevant levels of anxiety and 373 participants (28%) *possible *clinically relevant levels of depression. Adopting Snaith and Zigmond's [28] higher threshold for sensitivity of HADS-A and HADS-D scores of eleven or over, 204 participants (15%) demonstrated *probable *clinically relevant levels of anxiety and 161 participants (12%) *probable *clinically relevant levels of depression.

### Exploratory factor analysis

The Kaiser-Meyer-Olkin (KMO) measure of sampling adequacy and the Bartlett Test of Sphericity (BTS) were conducted on the data prior to factor extraction to ensure that the characteristics of the data set were suitable for the EFA to be conducted. KMO analysis yielded an index of 0.94, and BTS (χ^2 ^= 7758.34, df = 91, *p *< 0.001) was highly significant indicating the data satisfied the psychometric criteria for the factor analysis to be performed based on data distribution characteristics. Examination of individual item skew and kurtosis characteristics confirmed the suitability of the maximum likelihood factor extraction procedure [54]. Following extraction and oblimin rotation, two factors with eigenvalues greater than 1 emerged from analysis of the complete HADS and accumulatively accounted for 53% of the total variance. Factor loadings of individual HADS items in relation to the two-factor solution are shown in Table 2. Factor scores were calculated for each participant using regression and revealed the two extracted factors to be highly statistically and positively correlated, *r *= 0.82, *p *< 0.001, explaining 67% of the common variance between factors.

**Table 2:** Factor loadings of HADS items following maximum likelihood factor extraction with oblimin rotation

HAD Scale item		Factor 1	Factor 2
	*Anxiety sub-scale*		

(1)	I feel tense or wound up (AGI)	0.23	**0.45**
(3)	I get a sort of frightened feeling as if something awful is about to happen (ANX)	-0.02	**0.75**
(5)	Worrying thoughts go through my mind (ANX)	0.09	**0.69**
(7)	I can sit at ease and feel relaxed (AGI)	**0.35**	**0.33**
(9)	I get a sort of frightened feeling like 'butterflies' in the stomach (ANX)	-0.02	**0.72**
(11)	I feel restless as if I have to be on the move (AGI)	-0.01	**0.43**
(13)	I get sudden feelings of panic (ANX)	-0.02	**0.75**

	*Depression sub-scale*		

(2)	I still enjoy the things I used to enjoy	**0.95**	-0.14
(4)	I can laugh and see the funny side of things	**0.87**	-0.06
(6)	I feel cheerful	**0.72**	0.01
(8)	I feel as if I am slowed down	**0.38**	0.21
(10)	I have lost interest in my appearance	**0.40**	0.08
(12)	I look forward with enjoyment to things	**0.65**	0.09
(14)	I can enjoy a good book or TV programme	**0.42**	0.19

Note: Bold indicates that item loading on a factor is 0.30 or above.

**Figure 1:**
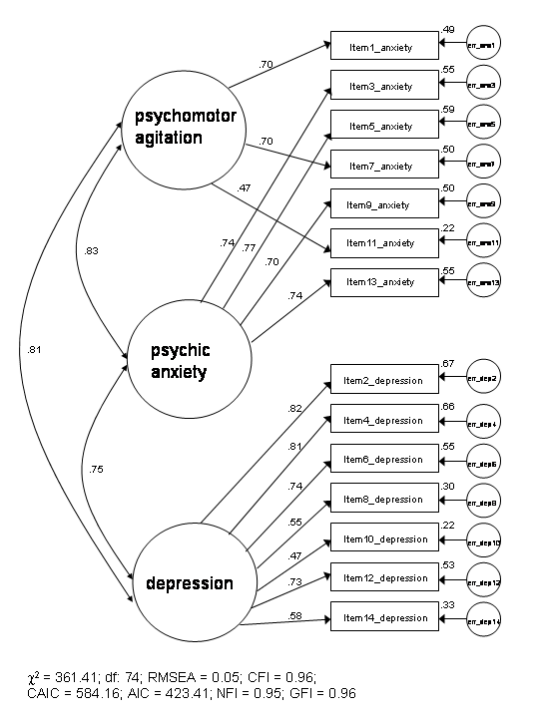
**Standardised factor loadings and between-factor correlations of the Friedman et al. [33] model. **Boxes represents HADS items labelled as shown as in table 2. Circles represents factors. One-way and two-way arrows indicate factor loadings and between-factor correlations, respectively.

Factors in final model in figure 1; AGI: psychomotor agitation; ANX: psychic anxiety; DEP: remains the same

### Confirmatory factor analysis

The factor models tested and accompanying fit indices are shown in Table 3. χ^2 ^goodness of fit analyses for all models were highly statistically significant (*p *< 0.001) indicating that a proportion of the total variance was unexplained by each model. Examination of the fit indices revealed that the best fit to the data was offered by Friedman et al.'s [33] three-factor model (see figure 1). This model provided consistently the best fit across all but one model fit assessment criteria. It was also found that both models of Dunbar et al.'s [32] three-factor model evaluation of Clark and Watson's [34] 'tripartite' model provided a 'best fit' to the data on a number of the fit indices tested (CFI, NFI and GFI) as did model 1 (CFI, NFI, and GFI) and model 2 (CFI, AIC, NFI and GFI) of Caci et al.'s [31] three-factor model. The two-factor models of Zigmond and Snaith [12] and Moorey et al. [43] offered poorer fits to the data compared to all three-factor models evaluated, however against accepted model fit convention, these two-factor models still offered an acceptable fit to the data. The single-factor model of Razavi et al. [44] was observed to offer the poorest fit to the data across all model fit estimates.

**Table 3:** Factor structure of the HADS determined by testing the fit of models derived from factor analysis. All χ^2 ^analyses were statistically significant at p < 0.01 (χ^2 ^degrees of freedom in parentheses).

Model	χ^2^	RMSEA	CFI	CAIC	AIC	NFI	GFI
Zigmond and Snaith (1983)	481.47(76)	0.06	0.95	718.84	539.47	0.94	0.95
Moorey et al. (1991)	480.77(76)	0.06	0.95	718.15	538.77	0.94	0.95
Caci et al. (2003) model 1	391.55(74)	0.06	**0.96**	645.30	453.55	**0.95**	**0.96**
Caci et al. (2003) model 2 *	352.02(62)	0.06	**0.96**	589.40	**410.02**	**0.95**	**0.96**
Dunbar et al. (2000) model 1	396.56(73)	0.06	**0.96**	658.49	460.56	**0.95**	**0.96**
Dunbar et al. (2000) model 2 ^#^	399.52(73)	0.06	**0.96**	661.45	463.52	**0.95**	**0.96**
Friedman et al. (2001)	361.41(74)	**0.05**	**0.96**	**584.16**	423.41	**0.95**	**0.96**
Razavi et al. (1990)	986.48(77)	0.09	0.88	1215.67	1042.48	0.87	0.88

Note: Bold indicates best model fit as a function of model fit index criterion.

* Three-factor model excludes item 10.

^# ^Hierachical arrangement of factors.

## Discussion

The findings of the current study offer a further important contribution to the evidence base regarding the underlying factor structure of the widely used HADS. It is worthy of note that high levels of HADS assessed anxiety and depression were observed in the study. This finding is consistent with investigators using this instrument in cardiac populations in other parts of the world [22,24,26] and verification of the need to screen for psychological disturbance in patients presenting with CHD.

The findings from the factor analyses conducted on the HADS data are of pertinent methodological as well as clinical interest. EFA of the HADS revealed two factors, the loadings of individual items being consistent with the anxiety and depression sub-scale domains. However, it was also observed that the HADS-A item-7 'I can sit at ease and feel relaxed' was jointly loading on both anxiety and depression factors, this split-loading slightly in favour of the depression factor. A recent EFA of the HADS conducted with patients with significant facial disfigurement [35] also revealed item-7 to be split-loaded between anxiety and depression latent domains. Martin and Newell [35] suggest that in circumstances of split-loading such as those observed in the current study, a two-factor solution may offer the most parsimonious solution in EFA, but may not provide the best identification of factors in terms of model fit. Martin and Newell's [35] rationale for this is that EFA is not a model evaluation technique, therefore identification of factors based on arbitrary cut-points such as eigenvalues and scree plots is likely to produce a psychometrically reductionist account of sophisticated relationships between observed and latent variables. Martin and Newell [35] proposed that the lack of apriori model specification in EFA provides a convincing psychometrically plausible explanation of inconsistencies between EFA and CFA in extracted factors and interpretation of data. Indeed, Martin and Newell [35] found a similar finding to that of the current investigation, EFA support for a two-factor model and CFA support for the superiority of three-factor compared to two-factor models. Interestingly, Dagnan et al. [55] and Mykletun et al. [56] identified three-factor initial solutions within the HADS but chose to dismise the third-factor, without a sound psychometric rationale. It is likely that an expectation of a presumed two-factor model makes it difficult to reconcile an unexpected three-factor model emerging from the data, therefore it is explainable why these researchers might choose to dismiss a third factor.

The findings from the CFA revealed the best model fit to be provided by Friedman et al.'s [33] three-factor mode (see figure 1). The 'next best' fit to the data is offered by Caci et al.'s [31] three-factor model 2. It was also observed that the remaining three-factor models tested [31,32] not only offered a good fit to the data but also provided a superior fit to the data compared to the two-factor models evaluated on a number of estimates of model fit. The two-factor models of Zigmond and Snaith [12] and Moorey et al. [43] did however offer an acceptable fit to the data. The uni-dimensional model of Razavi et al. [44] was found to offer a poor fit to the data, a finding consistent with previous research on the HADS across a variety of clinical groups [19,24,26,30,35]. There remains little doubt from the CFA analysis that the best fit to the data is offered by three-factor models irrespective of the clinical population from which the three-factor model was derived. The findings from the CFA have furnished compelling support of the HADS as a *tri-dimensional *instrument, consistent with contemporary research with this instrument across diverse clinical presentations [19,26,30,31,33,35].

## Conclusion

In conclusion, the current study found the German language version of the HADS to have an underlying three-dimensional factor structure following CFA in CHD patients, an observation consistent with UK [26] and Chinese [24] CHD populations. The traditional interpretation of the HADS as a two-factor (anxiety and depression) structure was also found to offer an acceptable fit to data, though inferior to that of the three-factor models. It can be concluded that the HADS may serve as useful screening purpose by being scored as two sub-scales of anxiety and depression. The clinical utilisation of the HADS continues to be invaluable in screening for mental disorders. Our results suggest that the assessment of the efficacy of interventions in evaluation studies by the HADS may be biased by problems in construct validity. Two decades have passed since the HADS was introduced to the clinical screening battery. The findings of this study and those of others, suggests that despite the clinical usefulness in screening the individual results of the HADS could be interpreted more precise in clinical routine. The differentiation of the anxiety scale in "psychomotor agitation" and "psychic anxiety" in the best fitting model may be helpful in the interpretation of individual results of patients. These results may improve our understanding of the process of adaptation in patients with somatic illness. A separate analysis of subscales in clinical trials may reduce bias caused by somatic medical conditions of the patients. Agitation might be more likely biased by the medical status of the patients.

## Authors' contributions

JB designed the study, carried out the data collection and clinical assessment. JB drafted part of the manuscript and was involved in the interpretation of the findings. CRM developed the statistical framework, carried out the statistical analysis and drafted part of the manuscript. Both authors have no competing financial or other interest in relation to this manuscript.

## Funding

The study was funded by the Federal Ministry of Education and Research, Germany; Regional Pension Insurance Institute, Baden-Wuerttemberg, Germany, LVA 02 804 and is part of the Rehabilitation Research Network South-West (Germany).
